# getphylo: rapid and automatic generation of multi-locus phylogenetic trees

**DOI:** 10.1186/s12859-025-06035-1

**Published:** 2025-01-18

**Authors:** T. J. Booth, S. Shaw, P. Cruz-Morales, T. Weber

**Affiliations:** The Novo Nordisk Foundation Center for Biosustainability, Danmarks Tekniske Universitet, Kongens Lyngby, Denmark

**Keywords:** Phylogenetics, Software, Evolution, Orthology, Taxonomy, Genomics

## Abstract

**Background:**

The increasing amount of genomic data calls for tools that can create genome-scale phylogenies quickly and efficiently. Existing tools rely on large reference databases or require lengthy de novo calculations to identify orthologues, meaning that they have long run times and are limited in their taxonomic scope. To address this, we created getphylo, a python tool for the rapid generation of phylogenetic trees de novo from annotated sequences.

**Results:**

We present getphylo (**Ge**nbank **t**o **Phylo**geny), a tool that automatically builds phylogenetic trees from annotated genomes alone. Orthologues are identified heuristically by searching for singletons (single copy genes) across all input genomes and the phylogeny is inferred from a concatenated alignment of all coding sequences by maximum likelihood. We performed a thorough benchmarking of getphylo against two existing tools, autoMLST and GTDB-tk, to show that it can produce trees of comparable quality in a fraction of the time. We also demonstrate the flexibility of getphylo across four case studies including bacterial and eukaryotic genomes, and biosynthetic gene clusters.

**Conclusions:**

getphylo is a quick and reliable tool for the automated generation of genome-scale phylogenetic trees. getphylo can produce phylogenies comparable to other software in a fraction of the time, without the need large local databases or intense computation. getphylo can rapidly identify orthologues from a wide variety of datasets regardless of taxonomic or genomic scope. The usability, speed, flexibility of getphylo makes it a valuable addition to the phylogenetics toolkit.

**Supplementary Information:**

The online version contains supplementary material available at 10.1186/s12859-025-06035-1.

## Background

Phylogenetic trees, or phylogenies, are fundamental to our understanding of evolution. Molecular phylogenies are visual representations of evolutionary relationships inferred from DNA or protein sequences [1–4]. Although species phylogenies can be inferred from single loci, such as ITS [5] or, 16S [6, 7] or 18S [8, 9] ribosomal RNA genes, this does not take advantage of the available genomic information and can lead to unreliable results [7, 9]. Therefore, it is common practice to use multiple genes to increase the number of informative sites and improve reliability [1–4]. However, selecting sequences for phylogenetic analysis is challenging because only orthologous sequences produce reliable topologies. In other words, evolutionary events, such as gene duplication or horizontal gene transfer, may make sequences unsuitable for inferring organism-level phylogenies [1]. As such, there has been significant effort to curate databases of orthologous sequences [10, 11]. Traditionally, these databases consist of a small number of well characterised sequences, typically intergenic spacers (e.g., ITS [12] or various plastid spacers [13]) or so-called ‘housekeeping’ genes (*atpD *[14], *rpoB *[14], *recA *[15] etc.).

Whole genome sequencing has enabled the construction of more robust phylogenies, owing to the increased number of genes available for analysis. However, manual curation of orthologous loci is time consuming, so tools such as autoMLST [2], GTDB-Tk [3], and TYGS [4], have been developed to automatically build trees from genomic input. These tools are useful for providing taxonomic classifications by helping to select reference genes and genomes, however they rely on predefined lists of genes or reference databases (up to 320 GB in the case of GTDB-Tk), or require lengthy de novo calculations, meaning that they can have long run times and are limited in their taxonomic scope (e.g. limited to bacteria and archaea in the case of GTDB-Tk).

Here, we present getphylo (**Ge**nbank **t**o **Phylo**geny), a tool that automatically builds phylogenetic trees from annotated genomes alone. Orthologues are identified heuristically by searching for singletons (single copy genes) across all input genomes. Trees are inferred from a concatenated environment by maximum likelihood as implemented in fasttree [16] or IQ-TREE [17]. getphylo has been designed to run quickly with low system requirements and without the need of additional databases. In addition, getphylo is flexible and can automatically generate high-quality phylogenies of not only genomes, but other genetic elements such as plasmids, prophages, or gene clusters.

## Implementation

**Fig. 1:**
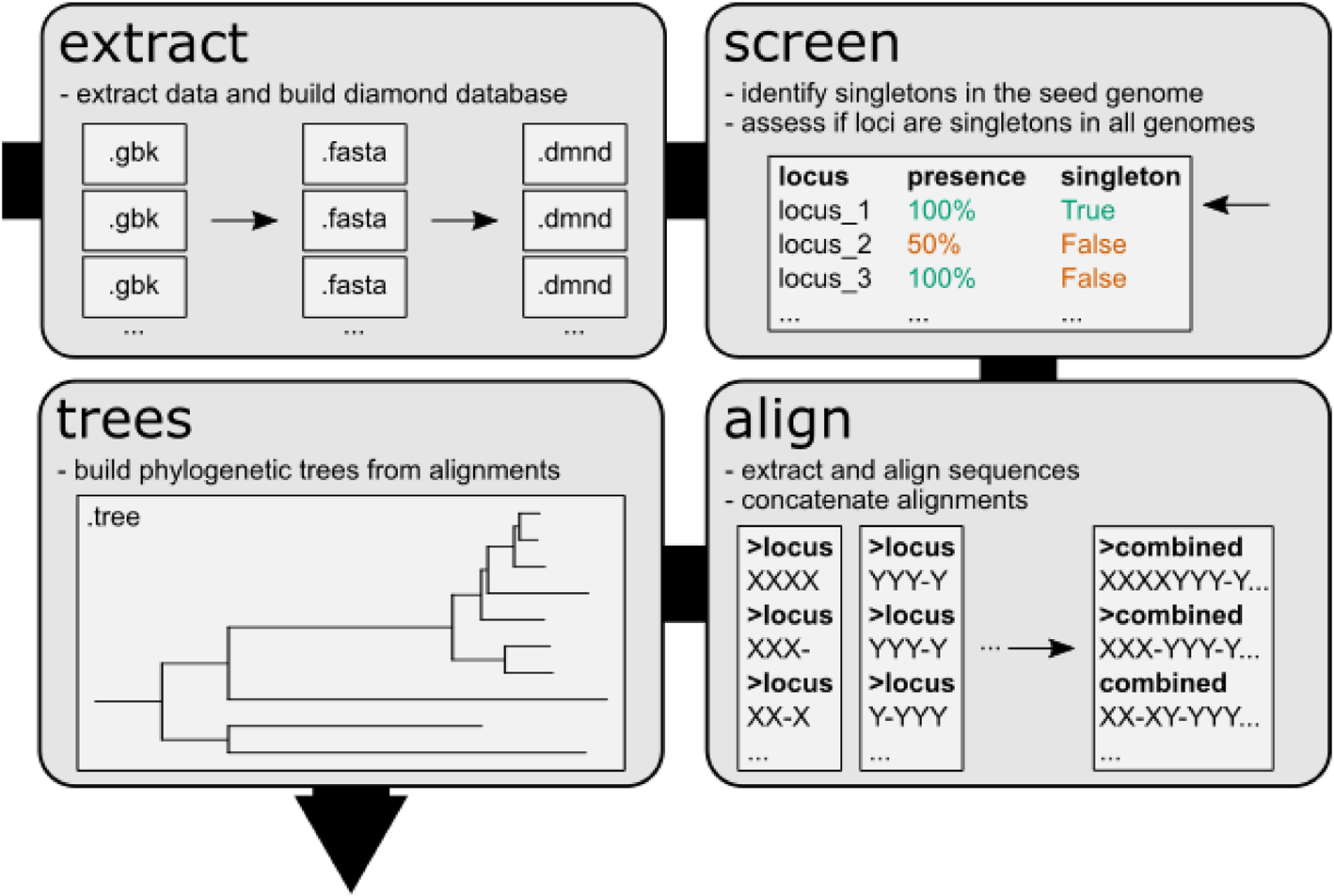
The workflow for getphylo. A simplified schematic of the getphylo’s modular workflow showing the functions of the extract, screen, align and trees modules

Getphylo is implemented in python (> = 3.7). Its dependencies are DIAMOND v0.9 [18], MUSCLE v3.8 [19], FastTree v2.1 [16], IQ-TREE [17] and Biopython 1.80 [20]. The package consists of four core modules that run sequentially: extract, screen, align and trees; a utility module (utils); and three dependency-specific modules (diamond, muscle and fasttree). An overview of the workflow is shown in Fig. 1.

First, the extract module extracts the protein coding sequences from each GenBank file and writes them as fasta files. By default, getphylo searches for ‘locus_tag’ annotations to account for standard GenBank inputs, but this can be redefined by the user using the --tag option. Once extracted, a DIAMOND database is built for each individual genome from the protein sequences.

The screen module then selects which sequences will be used for inferring the phylogeny. It identifies every singleton (genes with no homologues within the same genome) within a seed genome by performing an all vs. all blastp search using DIAMOND [18]. Each singleton is then queried against all the remaining genomes. If a given gene is present as a singleton in all genomes, it is considered orthologous and suitable for phylogenetic analysis. By default, sequences are only selected if they are present in all genomes. This threshold can be lowered using --presence, however this should be used with caution as this may introduce a significant amount of missing data into the alignments. The number of loci may also be limited using the --maxloci parameter, which will reduce runtime in cases where genomes are very closely related.

Next, the list of loci is passed to the align module that extracts the target sequences into separate fasta files. Each set of sequences is aligned independently using MUSCLE (both MUSCLE3 and MUSCLE5 are supported) [18, 19] and subsequently concatenated into a single alignment. Partition data and all individual alignments are provided by the align module for seamless integration into other phylogenetic workflows (e.g., model testing with IQ-TREE [17]).

Finally, the trees module uses FastTree 2 [16] or IQ-TREE 2 [17] to build phylogenies the combined alignment. Also, the --build-all flag will generate trees for each individual alignment so that their congruence of can be assessed. These trees can then be viewed in the user’s viewer of choice (e.g., iTOL [21]).

For convenience, getphylo employs a checkpoint system meaning that the analysis can be restarted from any step. This is particularly useful for building trees from proteomes, where GenBank files containing the nucleotide sequences may not be available. Many other parameters in getphylo can be adjusted to optimise performance. Full details can be found in the documentation and a comprehensive wiki (https://github.com/drboothtj/getphylo/wiki) has been written to help users with common tasks, questions, and feedback. Alternatively, getphylo may also be used in ‘quick start’ mode by simply navigating to a folder containing GenBank files and running the command ‘getphylo’ in the console.

## Results and discussion

### Benchmarking

We aimed to benchmark against four categories: job time, tree support, tree topology and tree consensus. Although no software offers a direct comparison to getphylo, similar functions are available in autoMLST [2] and GTDB-tk [3]. Both tools were developed primarily as taxonomic tools and therefore have many additional features (e.g. reference strain selection) that are not needed for comparison to getphylo. Therefore, in some cases, significant modification to the workflows was required to produce comparable results (for full details see Supplementary Information). Where possible, workflows were run with default parameters to avoid over-parameterisation. For benchmarking, we curated three datasets of 100 high quality *Streptomyces* genomes and three subsets consisting of 10 genomes from each of the larger datasets (Supplementary Figure S1). For more details on dataset curation, please see the Supplementary Information. Benchmarking data, including all output trees, are provided online (https://github.com/drboothtj/getphylo_benchmarking).

A direct comparison of job time is difficult because of the differences in workflows and the varying effects of sample size and diversity on algorithmic complexity across the three programs. However, for our datasets, job time for getphylo was lower across all runs (Table 1; Supplementary Figure S2).

**Table 1:** Benchmarking of getphylo. A comparison of getphylo, autoMLST and GTDB-tk All programs were run on random sets of 10 and 100 high quality (< 20 contigs; N50 > 1 Mb) *Streptomyces* genomes from the NCBI database. The time taken for each run and the normalised sum of the Robinson-Foulds distances (NSUMRF) are shown (8 vCPUs, 32GiB RAM, maximum frequency of 3.5 GHz). Full data is provided in the Supplementary Information

Method	Genomes	Time 10 loci, s	Time all loci,s (number of loci)	NSUMRF, 10 loci	NSUMRF, all loci
getphylo (This study)	10	19 ± 1	281 ± 27 (562 ± 51)	0.13	0.09
	100	164 ± 34	712 ± 59 (92 ± 8)	0.25	0.20
autoMLST (Alanjary et al.[2])	10	245 ± 23	759 ± 272 (325 ± 23)	0.11	0.09
	100	1816 ± 371	4430 ± 881 (157 ± 10)	0.27	0.21
GTDB-tk (Chaumeil et al.[3])	10	N/A	312 ± 84	N/A	0.11
	100	N/A	2429 ± 10	N/A	0.23

**Fig. 2:**
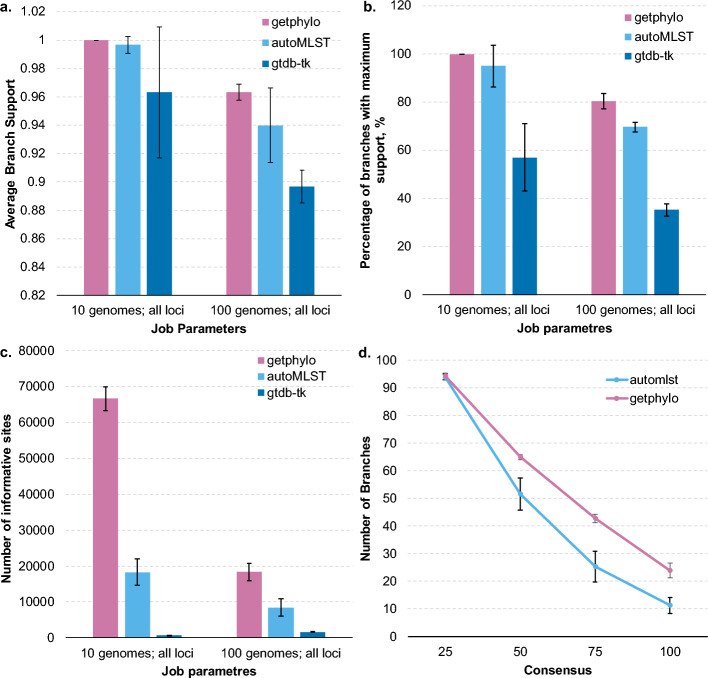
Benchmarking of getphylo, autoMLST and GTDB-tk. Comparison of automated phylogenetics pipelines getphylo (this study), autoMLST (Alanjary et al., 2019) and GTDB-tk (Chaumeil et al., 2020) when building the phylogenies of 100 *Streptomyces* genomes. The comparison shows information about branch support (**a** and **b**), the number of informative sites in the combined alignment (**c**), and the congruence of individual protein trees (**d**)

Across all datasets, getphylo produced the most well supported trees in terms of likelihood values (Fig. 2a; Supplementary Figure S3) and the percentage branches with maximum support (Fig. 2b; Supplementary Figure S4). It is important to note that this does not strictly mean that the tree more accurate, however it hints that the underlying sites are more congruent on average. Indeed, individual gene trees were consistently more congruent for loci selected by getphylo than those of autoMLST (note: due to GTDB-tk’s limitation on informative sites it could not be meaningfully tested) (Fig. 2d). Building consensus trees from fixed numbers of loci confirmed that this was not a sampling effect (Supplementary Figure S5). Furthermore, the sum of the Robinson-Foulds values between getphylo’s trees and all other trees were comparable or lower than those produced by the other workflows (Table 1; Figure S6; Table S1 and S2). This means that these trees were the least dissimilar to other trees in the dataset, i.e. the trees produced by getphylo are closest to the consensus between all three programs. In our benchmarking experiment, getphylo sampled more loci and informative sites on average (Fig. 2c; Figure S7), which may have contributed to its superior performance.

Finally, since getphylo offers to use both FastTree [16] and IQ-TREE [17] for tree construction, we decided to compare the effect of model testing on the on final tree topology and run time. The resulting trees were identical. Given that using IQ-TREE resulted in a huge increase in run time, we advise that FastTree be used for preliminary runs and when using the --build-all command. IQ-TREE should be used when only constructing the combined tree.

### Case studies

Our goal was to create a flexible workflow capable of producing multi-locus phylogeny, independent of taxonomic group or genetic scale. To demonstrate the flexibility of getphylo, we analysed four additional datasets representing a broad range of potential applications (Supplementary Information: Case Studies 1—4).

**Fig. 3:**
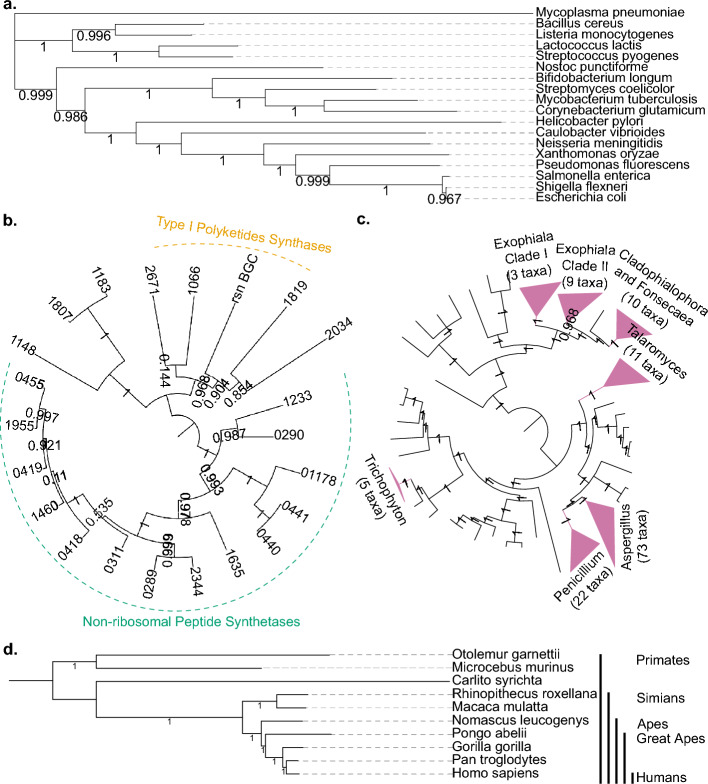
getphylo test cases. Four case studies were used to demonstrate the flexibility of getphylo. It generated phylogeny of the following genomic datasets: **a** 18 bacterial genomes from across the domain; **b** 22 biosynthetic gene clusters based on conserved proteins for 3,5-dihydroxybenzoic acid biosynthesis (rooted at midpoint, major polyketide synthase and non-ribosomal peptide clades are shown; taxa are signified by truncated MiBiG[26] identifiers with the format BGC00XXXX); **c** 165 Eurotiomycete genomes (major clades are collapsed for display purposes, branches with < 1 branch support have been collapsed into polytomies) and; **d** 10 primate genomes (rooted at *Otolemur garnettii*). Detailed trees are available in the Supplementary Information and online

Firstly, we wanted to test how getphylo performed on very broad taxonomic groups. We analysed a sample of 18 bacteria, representative of the diversity of the domain (Supplementary Information Case Study 1). From these genomes, getphylo identified 12 proteins representing 3,685 informative sites. The analysis was completed in 36 s (8 vCPUs, 32GiB RAM, maximum frequency of 3.5 GHz) and the resulting tree is shown in Fig. 3a (Supplementary Figure S8). The tree was well supported (0.996 average branch support and 66% of branches showing maximum support) and shows the expected topology [22]. The loci identified by getphylo consisted largely of known orthologous groups, including classical ‘housekeeping’ genes, such as *rpoB *[14] and various ribosomal proteins (Supplementary Table S3). This demonstrates the ability of getphylo to identify orthologues de novo, quickly and reliably across diverse genomes.

Next, we wanted to demonstrate the flexibility of getphylo to analyse other genetic elements (Supplementary Information; Case Study 2). Multi-locus phylogenies are most often used to build genome-scale phylogenies, but they are also useful for examining other elements such as phages and prophages, genomic islands, plasmids and gene clusters (e.g. [23] and [24]). These elements may share distinct evolutionary histories when compared to their hosts and the ability to provide a quick, initial overview of their relationships is extremely valuable [24]. To demonstrate this function, we reconstituted the evolutionary history of the resorculin biosynthetic gene cluster (*rsn* BGC) [25] (Case Study 2; Supplementary Figure S9). The resorculins are bacterial metabolites produced by *Streptomyces* sp. MST-91080. Their biosynthesis is encoded by a 10-gene BGC. 218 BGCs sharing 3 genes or more with *rsn* were identified from MiBiG 3.1[26] using cblaster v. 1.3.18 [27]. getphylo successfully identified the conserved genes for 3,5-dihydroxybenzoic acid biosynthesis, in line with our previous study [25] and built a corresponding phylogeny from the 22 BGCs that contained homologues for RsnE and RsnF (Fig. 3b; Supplementary Figure S9, 10). The resulting tree neatly showed the expected relationship between the glycopeptides and other 3,5-dihydroxybenzoic acid derived natural products. This demonstrates the flexibility of getphylo to build phylogeny from other genetic scales through the identification of conserved sub-clusters.

Next, to assess how getphylo handles eukaryotic genomes, we used getphylo to construct phylogenies of primates (Case Study 3) and fungi (Case Study 4). For the primate tree, we analysed 10 genomes. Despite the large size of the genomes, (2.4 – 3.6 Gb), the analysis concluded relatively quickly, taking only 18 min 53 s. The resulting tree (Fig. 3d; Supplementary Figure S11) showed maximum possible support and is congruent with previously published phylogenies [28–30]. The alignment consisted of 82 concatenated coding sequences. For comparison, Vanderpool et al. [30] identified 1,730 genes existing as single copy orthologues across a larger selection of primates using blastp homology searching combined with Marcov Clustering. This result highlights the speed of getphylo, even when analysing even large genomes, and the loss of potentially informative sites resulting from getphylo’s strict criteria. This trade-off is discussed in detail below.

For the fungal tree, we collected a dataset of 165 Eurotiomycete proteomes. The initial analysis identified 362 loci representing 316,156 informative sites, however due to memory limitations with FastTree (16 Gb RAM), the analysis could not be completed. Repeating the analysis using the –maxl flag limited the analysis to 100 loci and 87,002 informative sites. The resulting tree (Fig. 3c; Supplementary Figure S12) had and average branch support of 0.89 with 88% branches showing maximum support and was congruent with a recently published genome-scale phylogeny [31]. As existing tools are tailored towards bacterial and archaeal genomes, we believe getphylo will be particularly useful for exploring eukaryotic genomes, especially fungal where substantial data are available.

### Strengths and limitations

Fundamental to the creation of species-level phylogeny is the selection of orthologous sequences [10, 32]. In the genomic era, the automation of this process is a necessary step, but there is an understandable concern that these tools are subject to limitations and may provide misleading results if used carelessly. It is important that end-users understand the output of their analysis and the limitations of their chosen methods. To this end, we: i) implemented getphylo to provide comprehensive output for each stage in its pipeline; ii) have performed thorough benchmarking to demonstrate the relative performance of getphylo; and iii) provide a detailed discussion on the strengths and limitations of our approach.

At the core of getphylo is its algorithm for detecting orthologues. The difficulty of identifying orthologues is well documented [32, 33]. The dynamism of the evolutionary process means that homologous sequences are not necessarily orthologous. For example, homology may result from gene duplication (paralogues) as opposed to speciation. Gene duplication, horizontal gene transfer, hybridization and introgression are all examples of evolutionary processes that confound the identification of orthologues. As a result, there exists no single method for orthology detection and orthology can only ever be inferred. A consensus has emerged that the approach should be tailored to the specific demands of the analysis (e.g. number of sequences, computational resources) [32].

The most commonly used method to find orthologues is to search for reciprocal best hits (RBHs) within a dataset. This is advantageous as it allows the separation of orthologues with potential duplicates or other homologues however it is computationally demanding. In contrast, getphylo defines genes as orthologues only if they exist as singletons in all genomes (Supplementary Figure S13-14). This means that all hits are definitionally RBHs and they are identified with exponentially fewer calculations. The tradeoff is that genes with any other homologues within the same genome are discarded. This means that in larger genomes with more homologous sequences, potentially informative data will be discarded (see Case Study 3). However, orthologue clustering, which is time consuming and difficult to optimize, can be skipped entirely resulting in a workflow that is exponentially faster. The Case Studies demonstrate (Fig. 2) that this tradeoff is acceptable for a fast and flexible. As with all methods, there are scenarios that can confound this algorithm. For example, including a large number of incomplete genomes may result in the misidentification of orthologues. However, this problem applies to all RBH-based methodologies. In fact, it is significantly less likely in getphylo as the duplicate gene would have to be absent in all genomes and the data would be discarded. Nevertheless, we recommend removing low quality assemblies before analysis with getphylo.

We have provided benchmarking between getphylo and two contemporary, automated, phylogenetics workflows: autoMLST [2] and GTDB-tk [3]. As we have demonstrated, getphylo offers some unique advantages, chiefly its low job time and flexibility offered by the de novo identification of orthologues. However, unlike autoMLST, it does not offer the selection of reference genomes or a web-portal. Additionally, GTDB-tk samples relatively few sites as it was designed to build phylogenies of 10,000 s of genomes. getphylo is unlikely to be able to handle datasets of this size due to the likelihood of genes being duplicated in at least one genome. Each of these tools have specific use cases, but it is important to highlight the importance of cross validation and getphylo’s unique workflow makes it a valuable tool for this purpose. It is important to reiterate that manual curation and examination of loci is crucial when testing specific evolutionary hypotheses, especially when studying sexually reproducing species and in instances where gene trees may not be reprisentative of species trees, e.g. at a population level where recombination among individuals may be high. To this end, we have aimed to make the intermediate results of getphylo readily available so its results can be easily evaluated. This includes providing all individual gene trees (on request) so that their congruence can be assessed. For the time being, getphylo only infers trees from the concatenated alignment, however, as individual gene trees and alignments are provided, the user can easily infer trees via other methods.

## Conclusions

We have developed getphylo, a quick and reliable tool for automating the generation of genome-scale phylogenetic trees. We have demonstrated that getphylo can produce phylogenies comparable to other software in a fraction of the time and without the need for storing local databases of reference genes. getphylo’s ‘strict heuristic’ workflow means that it can rapidly identify orthologues from a wide variety of datasets regardless of taxonomic scope. As getphylo uses a unique methodology to produce genome-scale phylogenies, it can also serve as a valuable second metric for cross-validating existing methods. The usability, speed, flexibility of getphylo makes it a valuable addition to the phylogenetics toolkit.

## Materials and methods

Getphylo is implemented using python 3.7. It also requires the installation of DIAMOND v0.9 [18], MUSCLE [19] version 3 or 5, FastTree v2.1 [16], IQ-TREE 2.3.0 [17] and Biopython 1.80 [20]. Benchmarking was performed against autoMLST as implemented in the autoMLST-simplified-wrapper (revision 0df6094) and GTDB-tk version 2.1.1. Detailed information about the modifications to these workflows is available as supplementary material. For analysis of the resorculins the original dataset was curated from MiBiG version 3.1 [26] and cblaster version 1.3.18 [27].

All genomes were obtained from the NCBI. Accessions, resulting trees and benchmarking data are available at https://github.com/drboothtj/getphylo_benchmarking.

## Supplementary Information

Additional file 1.

## Data Availability

Getphylo is freely available and is downloadable through the Python Package Index (pip install getphylo; https://pypi.org/project/getphylo/) and GitHub (https://github.com/drboothtj/getphylo). The example data described in this manuscript and the sample outputs are also available on GitHub (https://github.com/drboothtj/getphylo_benchmarking). A user guide can be found at: https://github.com/drboothtj/getphylo/wiki. Project name: getphylo. Project home page: ht tp s://gith ub.co m/drb oothtj/ge tphylo. Operating system(s): Linux. Programming language: Python. Other requirements: BioPython, DIAMOND, FastTree 2, IQ-TREE 2, and MUSCLE3 or MUSCLE5. License: GNU General Public License version 3. Any restrictions to use by non-academics: No, see license.

## Abbreviations

autoMLST: Automated multi-locus species tree
CPUs: Central processing units
DIAMOND: Double index alignment of next-generation sequencing data
FASTA: FAST-all
Getphylo: GenBank to phylogeny
GHz: Gigahertz
GiB: Gibibyte
GTDB-tk: Genome taxonomy database tool kit
ITS: Internal transcribed spacer
IQ-TREE: IQPNNI and TREE-PUZZLE software
MUSCLE: Multiple sequence comparison by log-expectation
RAM: Random access memory
TYGS: Type genome analysis server
